# Cryo-electron Microscopy Structures of Chimeric Hemagglutinin Displayed on a Universal Influenza Vaccine Candidate

**DOI:** 10.1128/mBio.00257-16

**Published:** 2016-03-22

**Authors:** Erin E. H. Tran, Kira A. Podolsky, Alberto Bartesaghi, Oleg Kuybeda, Giovanna Grandinetti, Teddy John Wohlbold, Gene S. Tan, Raffael Nachbagauer, Peter Palese, Florian Krammer, Sriram Subramaniam

**Affiliations:** a Center for Cancer Research, National Cancer Institute, National Institutes of Health, Bethesda, Maryland, USA; b Department of Microbiology, Icahn School of Medicine at Mount Sinai, New York, New York, USA; c Graduate School of Biomedical Sciences, Icahn School of Medicine at Mount Sinai, New York, New York, USA; d Faculty of Life Sciences, University of Vienna, Vienna, Austria; e Department of Medicine, Icahn School of Medicine at Mount Sinai, New York, New York, USA

## Abstract

Influenza viruses expressing chimeric hemagglutinins (HAs) are important tools in the quest for a universal vaccine. Using cryo-electron tomography, we have determined the structures of a chimeric HA variant that comprises an H1 stalk and an H5 globular head domain (cH5/1 HA) in native and antibody-bound states. We show that cH5/1 HA is structurally different from native HA, displaying a 60° rotation between the stalk and head groups, leading to a novel and unexpected “open” arrangement of HA trimers. cH5/1N1 viruses also display higher glycoprotein density than pH1N1 or H5N1 viruses, but despite these differences, antibodies that target either the stalk or head domains of hemagglutinins still bind to cH5/1 HA with the same consequences as those observed with native H1 or H5 HA. Our results show that a large range of structural plasticity can be tolerated in the chimeric spike scaffold without disrupting structural and geometric aspects of antibody binding.

**Importance** Chimeric hemagglutinin proteins are set to undergo human clinical trials as a universal influenza vaccine candidate, yet no structural information for these proteins is available. Using cryo-electron tomography, we report the first three-dimensional (3D) visualization of chimeric hemagglutinin proteins displayed on the surface of the influenza virus. We show that, unexpectedly, the chimeric hemagglutinin structure differs from those of naturally occurring hemagglutinins by displaying a more open head domain and a dramatically twisted head/stalk arrangement. Despite this unusual spatial relationship between head and stalk regions, virus preparations expressing the chimeric hemagglutinin are fully infectious and display a high glycoprotein density, which likely helps induction of a broadly protective immune response.

## IMPORTANCE

Chimeric hemagglutinin proteins are set to undergo human clinical trials as a universal influenza vaccine candidate, yet no structural information for these proteins is available. Using cryo-electron tomography, we report the first three-dimensional (3D) visualization of chimeric hemagglutinin proteins displayed on the surface of the influenza virus. We show that, unexpectedly, the chimeric hemagglutinin structure differs from those of naturally occurring hemagglutinins by displaying a more open head domain and a dramatically twisted head/stalk arrangement. Despite this unusual spatial relationship between head and stalk regions, virus preparations expressing the chimeric hemagglutinin are fully infectious and display a high glycoprotein density, which likely helps induction of a broadly protective immune response.

## INTRODUCTION

Influenza causes substantial morbidity and mortality, with hundreds of millions of infections occurring annually worldwide (1). Current vaccine formulations predominantly rely on eliciting neutralizing antibodies that target the highly variable head domain of the surface-expressed viral glycoprotein hemagglutinin (HA) and are generally exclusively effective against infectious viral populations that match the vaccine strain (2–4). For this reason, new vaccines need to be created almost every year as the circulating influenza virus strains mutate rapidly (5). The slow and costly production of these vaccines renders their use problematic, especially in the case of a sudden pandemic, when seasonal vaccines are unlikely to be effective. Efforts to create a universal vaccine, which would obviate a yearly vaccine, are ongoing and include strategies that attempt to boost the population of antibodies that target the more highly conserved stalk domain of HA (reviewed in references  6, 7, and 8).

One such strategy uses constructs that express chimeric HA proteins, which combine stalk and head domains from different HA subtypes. After an initial exposure to vaccine or infection, subsequent boosters with chimeric HA constructs expressing conserved stalk domains in combination with different head domains are given (9–11). Sequential exposure to chimeric HA proteins that express the same stalk domain but varied head domain subtypes redirects the immune response toward the immuno-subdominant conserved stalk domain (12). This strategy results in an increased production of broadly protective stalk-targeting neutralizing antibodies, which are rarely produced after vaccination with seasonal inactivated influenza virus vaccines (13, 14). Vaccination with these constructs has been successful in animal models, including mice (12, 14) and ferrets (15), and plans are under way to use these viruses in human clinical trials. One important criterion for any universal influenza vaccine candidate is that it should maintain the epitopes necessary for protection by neutralizing antibodies. At present, no three-dimensional (3D) structural information is available on these recombinant HA proteins either in native form or when they are bound to neutralizing antibodies. This is an especially important gap to fill because neutralizing stalk-reactive antibodies are thought to bind almost exclusively to fragile, conformational epitopes (16–20). The presence of correctly folded HA stalk domains in the vaccine is crucial to elicit neutralizing antibodies against these conformational epitopes. Using cryo-electron tomographic methods we developed previously for structure determinations of native viral glycoproteins (21–23), we present here a comparative structural analysis of cH5/1 HA, a chimeric HA protein that is comprised of H1 stalk and H5 head domains, with the corresponding nonchimeric H1 and H5 variants, each expressed on the viral surface.

## RESULTS AND DISCUSSION

### Chimeric HA shows structural differences compared to H1 and H5 HA.

**FIG 1:**
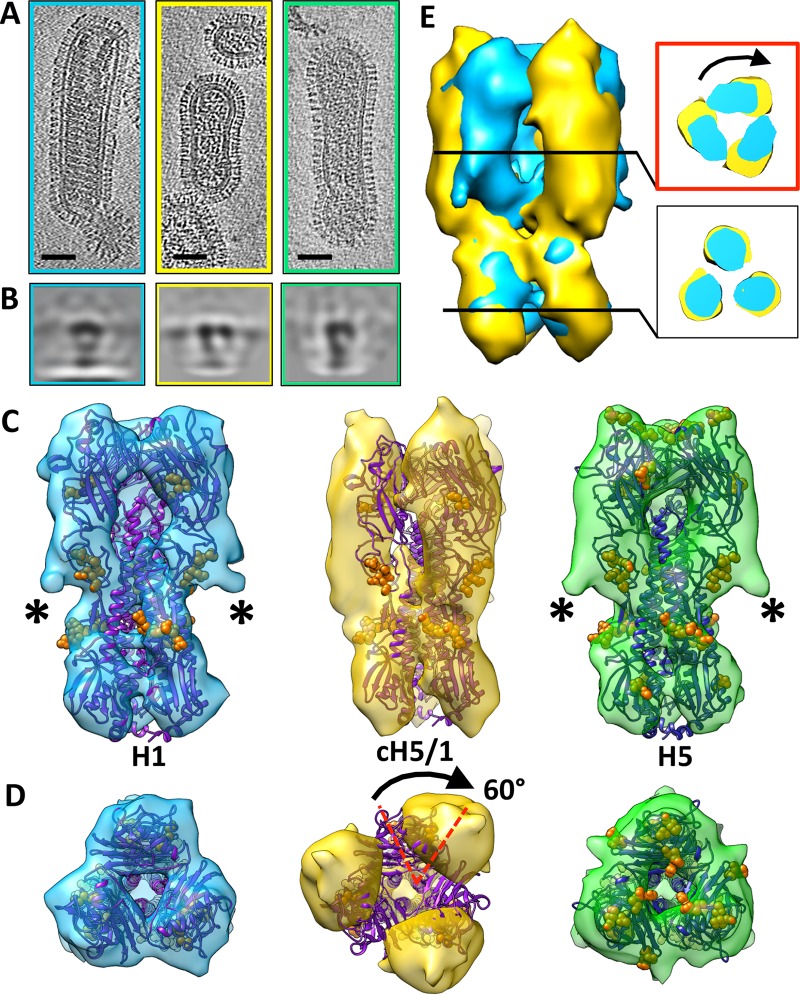
Cryo-EM density maps reveal differences in morphology of the chimeric HA compared to H1 and H5 HA. For all panels, H1 HA, cH5/1 HA, and H5 HA are indicated by cyan, gold, and green, respectively. (A) Tomographic slices of single pH1N1, cH5/1N1, or H5N1 viruses are shown from left to right, respectively. (B) Projection views of slices through averaged subtomogram volumes for (from left to right, respectively) H1, cH5/1, or H5 HA show a side view of each HA. (C and D) Isosurface representations of density maps are shown in side (C) and top (D) views, respectively. Density maps are fitted with X-ray coordinates for H1 HA (purple) or H5 HA (dark blue). Glycosylated residues are represented as solid orange spheres. Corresponding density within the density maps is marked by asterisks. (D) The mismatch between the X-ray coordinates and the cH5/1 HA density map is indicated with a black arrow. (E) An overlay of the cH5/1 and H1 HA maps aligned by the stalk region is shown. Cross-sectional views of the head and stalk regions (boxed insets) show the head region misalignment of the two maps (indicated by a black arrow).

**FIG 2:**
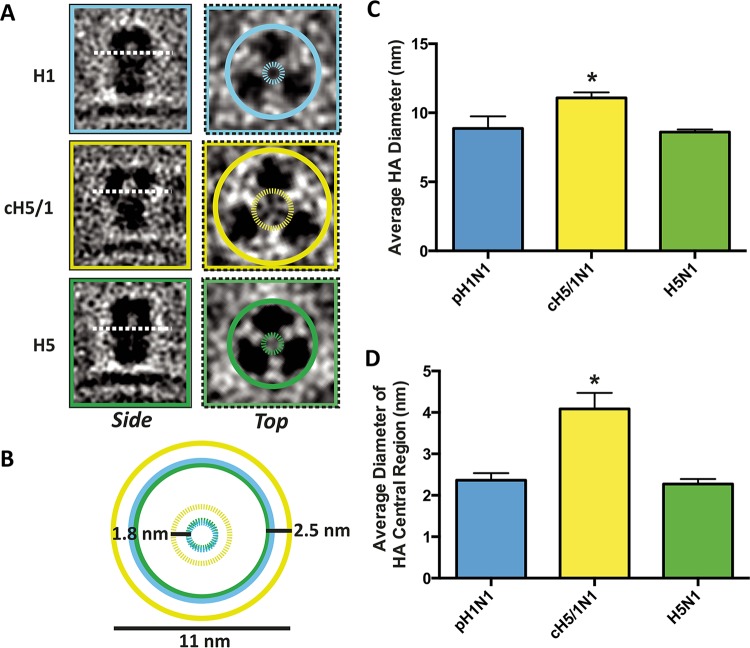
The chimeric HA spike has a more open structure than H1 or H5 HA. (A) Side- and top-view slices through subtomogram averages of HA are shown for H1 (cyan), cH5/1 (gold), and H5 (green) HAs. In the top-view images, solid circles are drawn around the perimeter of each glycoprotein, and dashed circles indicate the space between monomers in the central cavity of the spike. (B) Solid and dashed circles are shown superimposed without the subtomogram images. Values shown indicate the size of the largest gold circle (11 nm), the change in average spike diameter between cH5/1 and H1 or H5 HA (2.5 nm), and the change in average diameter of the inner spike cavity between cH5/1 and H1 or H5 (1.8 nm). (C and D) Subtomogram class averages for each HA protein were used to determine the diameter of HA at its widest point (C) and the diameter of the open space in the central cavity of the spike (D). Measurements are averaged from seven subtomogram classes, and values are shown as means plus standard errors of the means (SEM) (error bars). Values that are significantly different from the other values (*P* ≤ 0.01) as determined by one-way ANOVA are indicated by an asterisk. For each panel, HA type is indicated in cyan (H1), gold (cH5/1), or green (H5).

Viruses displaying either H1 (pH1N1), H5 (H5N1), or cH5/1 (cH5/1N1) HA (Fig. 1A) were analyzed using cryo-electron microscopy (cryo-EM) tomography and subvolume averaging, resulting in HA density maps that revealed differences in cH5/1 HA structure compared to native H1 or H5 HA (Fig. 1). Native H1 and H5 HA globular head monomers are more closely associated than cH5/1 HA monomers (Fig. 2). Compared to H1 or H5, cH5/1 HA shows a wider head domain (increase in width of ~25 Å at the apex of the spike) and greater separation between monomers (~20-Å-wider inner cavity) (Fig. 2), suggesting that the structure of chimeric HA is more open than that of H1 or H5. This observation was confirmed in two-dimensional (2D) projection views of subvolume class averages, which show a larger diameter in the inner spike cavity of cH5/1 compared with the closely associated monomers in the H1 and H5 head domains (Fig. 1B). Comparison of the structure of chimeric HA with the corresponding nonchimeric variants reveals a striking difference in HA morphology (Fig. 1C and D). A rotation of ~60° occurs between the stalk and head domains of cH5/1 HA, which is apparent from the misalignment of the H1 HA X-ray structure when fitted to the cH5/1 density map (Fig. 1C and D). This rotation is evident after alignment of the stalk domains of cH5/1 HA to HA from either H1 (Fig. 1E) or H5 (see Fig. S1A in the supplemental material). Cross sections taken at the head and stalk regions of these alignments show an ~60° rotation of the cH5/1 HA head domain in comparison to the native HA structures (inset panels of both Fig. 1E and Fig. S1A), while cross sections taken of an overlay between H1 and H5 HA proteins show a close fit in both the stalk and head domains (Fig. S1B, inset panels). Despite this dramatic difference in HA architecture, cH5/1 HA remains a functional protein and is able to induce production of broadly neutralizing antibodies in host organisms (12, 14, 15). In addition, the growth properties of viruses that express the chimeric HA proteins are similar to those of viruses expressing native HA, and these viruses are able to fuse with cells *in vitro* (9, 24), suggesting that these chimeric HA proteins are functional on the viral surface.

### cH5/1 HA density on the viral surface is significantly greater than that of H1 or H5 HA.

**FIG 3:**
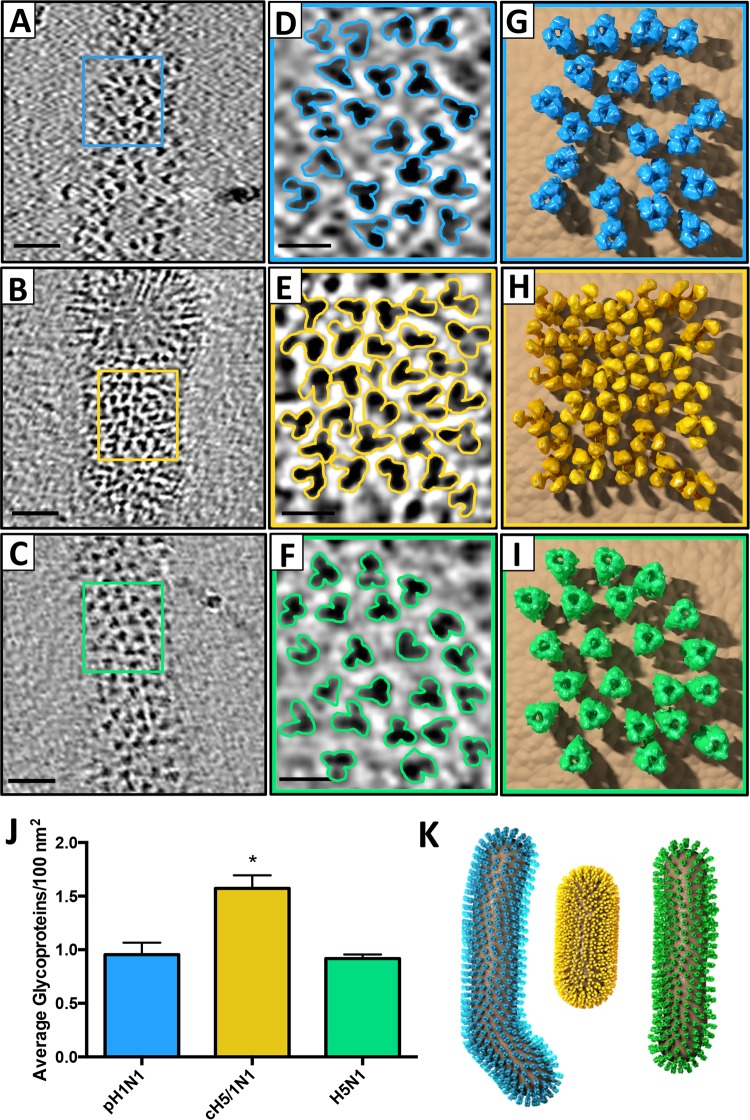
cH5/1 HA density on the viral surface is significantly greater than that of H1 or H5 HA. (A to C) HA trimers on the viral surface are shown in tomographic slices of pH1N1 (A), cH5/1N1 (B), and H5N1 (C). (D to F) Enlarged views of the boxed regions in panels A to C are shown in panels D to F with trimeric HA proteins outlined. (G to I) Models corresponding to the viral surface areas shown in panels D to F of pH1N1 (G), cH5/1N1 (H) and H5N1 (I) are shown with the respective HA density maps obtained by subvolume averaging. (J) HA trimer density per 100 nm^2^ plus standard error of the mean (SEM) of viral surface area is plotted for each influenza strain. The value that is statistically significantly different (*P* = 0.002) from the other values as determined by one-way ANOVA is indicated by an asterisk. (K) 3D virus models for pH1N1 (cyan), cH5/1N1 (gold), and H5N1 (green) were created based on spike density measurements.

Inspection of tomograms shows that cH5/1 HA glycoproteins are more densely packed on the viral surface than either H1 or H5 glycoproteins (Fig. 3A to C). cH5/1 HA proteins are more clustered and are in relatively close contact (Fig. 3E and H) in contrast to H1 (Fig. 3D and G) and H5 (Fig. 3F and I) proteins, which are more dispersed on the viral surface. Interspike distance between HA proteins on each viral strain was calculated, verifying that cH5/1 HA proteins are significantly closer together on the viral surface than H1 or H5 HA (see Table S1 in the supplemental material). The number of visible trimeric spikes in tomographic slices was determined to quantify the HA spike density on each viral strain (Table S1). When the spike densities for each strain were normalized to the number of spikes in a 100-nm^2^ area, HA spike density was significantly higher for the cH5/1N1 virus than for the pH1N1 or H5N1 strains (Fig. 3J; Table S1). On a model filamentous virus, these density measurements would correspond to 3,213 HA spikes per cH5/1N1 virus compared to 1,975 and 1,691 spikes per virus for pH1N1 and H5N1, respectively (Table S1). From these measurements, 3D virus models were created for each strain and are shown with the corresponding HA structures modeled on the viral surface (Fig. 3K).

The higher HA density on the chimeric viral surface could potentially be driven by interspike HA interactions caused by the unusual structure of the chimeric HA (cHA). We did not observe obvious HA-HA interactions in tomogram slices of the chimeric virus that could prove this hypothesis (Fig. 3E). However, we cannot rule out the possibility that interactions of this kind influence the formation of new virions and the arrangement of HA on the viral surface. All three viral strains showed highly pleomorphic shapes. Within each data set, we observed viruses that were small or large spheres as well as long or short filaments. The cH5/1N1 virus showed a trend toward shorter filaments compared to pH1N1 or H5N1, as shown in our 3D model (Fig. 3K). However, this difference was not found to be significant (see Table S1 in the supplemental material). Within each viral strain, the majority of virions were filaments between 100 and 200 nm long.

Neuraminidase (NA) tetramers are present on the viral surfaces of all viruses tested but were not visible in tomogram slices, perhaps due to their relatively low density in comparison with HA trimers. pH1N1 viruses display an NA/HA ratio of 1:5.5 compared to a ratio of 1:8.6 for cH5/1N1 viruses. While the number of visible spikes on the cH5/1N1 viral surface is increased compared to the native spike number, the ratio of NA shows a decrease in density on the chimeric strain compared to the native strain. Therefore, the increase in spike density is likely to be attributed to an increase in the number of HA proteins on the virus. At present, we cannot evaluate the significance of the higher density on the viral membrane, but it is conceivable that it may have an impact on better signaling through low-affinity B-cell receptors.

### H1- and H5-specific HA head-targeting antibodies bind in opposite orientations.

**FIG 4:**
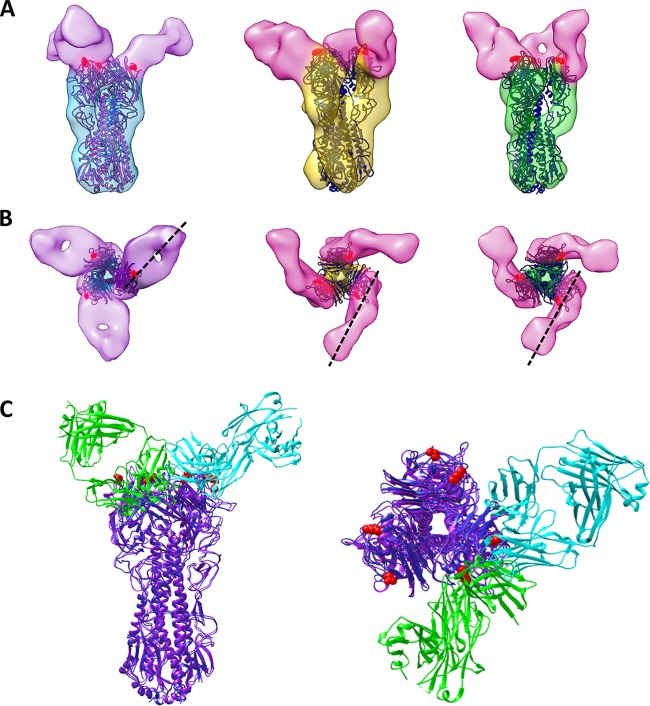
Structures of HA bound to head-binding antibodies reveal differences in antibody orientation. (A) Isosurface representations of H1 (cyan), cH5/1 (gold), and H5 (green) HAs bound to antibody 7B2 (purple) or 3F5 (pink) are shown superimposed with the corresponding crystal structures (H1 in purple and H5 in dark blue). Escape mutant residues are indicated by solid red spheres. (B) The black dashed lines show the directionality of 7B2 and 3F5 antibody binding from congruent monomers. (C) H1 and H5 crystal structures were fitted to their respective density maps, and X-ray coordinates from a representative head-binding antibody (PDB ID 4FQL) were fitted to either 7B2 (cyan) or 3F5 (green) map density.

In order to elucidate whether the rotational twist in the chimeric HA protein alters its ability to bind neutralizing antibodies, we determined structures of HA on pH1N1, cH5/1N1, or H5N1 viruses preincubated with antibodies that are expected to bind either the stalk or head regions of HA. Chimeric cH5/1 HA retains its ability to bind head-reactive antibodies, despite the difference in its morphology (Fig. 4). A comparison of the structures of H5 and cH5/1 HA bound to an H5-specific antibody, 3F5, revealed similarities in antibody binding orientation on the H5 head domains (Fig. 4A and B, pink antibodies). In contrast, the structure of H1 HA bound to an H1-specific antibody, 7B2, revealed that 7B2 binds HA in a different orientation compared to 3F5 (Fig. 4A and B, purple antibodies). A model was constructed to highlight the distinct binding locations and orientations of the 7B2 and 3F5 antibodies using a representative influenza head-binding Fab (4FQL) which was fitted to map densities corresponding to either 7B2 (cyan) or 3F5 (green) on one congruent monomer of H1 and H5 HA (Fig. 4C). To ascertain binding epitope regions of 7B2 and 3F5, the locations of escape mutations that result in loss of antibody binding were determined (see Table S2 in the supplemental material). These epitopes are consistent with the location of antibody density in our structures (Fig. 4, see red highlighted residues). Interestingly, the 50% inhibitory concentration (IC_50_) of 3F5 for cH5/1N1 viruses is 100-fold lower than for H5N1 viruses (Table S3) despite similar binding affinities for each protein (Table S4), suggesting that the more-open cH5/1 HA protein conformation may aid in more-effective neutralizing antibody protection. In principle, it is possible that the chimeric proteins may be less stable, but this seems unlikely given that viruses expressing the chimeric construct grow normally, are able to infect cells, and induce production of broadly neutralizing antibodies in host organisms.

We observed a viral aggregation phenotype evident in all viral preparations bound by head-specific antibodies (see Fig. S4 in the supplemental material). Aggregation occurs to the greatest extent in the cH5/1N1 (Fig. S4B and S4E) and H5N1 (Fig. S4C and S4F) samples incubated with 3F5 antibody, but it is also present in pH1N1 preparations incubated with 7B2 antibody (Fig. S4A and S4D), suggesting that aggregation is not dependent on the conformational state of HA. The viral aggregation observed is likely to be the result of interviral cross-linking by antibodies, as aggregates of protein A gold particles, which have an affinity for the Fc region of antibodies, localized to the same areas as the viral aggregates (Fig. S4D to S4F). The aggregation phenotype was not observed when virus was incubated in the presence of stalk-binding antibodies, 6F12 (cH5/1 and H1) or KB2 (9) (H5) (Fig. S4G to S4I). Antibody cross-linking of cH5/1N1 virions by 3F5 is visible in a tomogram slice, where additional density is present in between the glycoprotein spikes of adjacent viruses (Fig. S4J, red box), but not in tomogram slices of cH5/1N1 virus incubated with the stalk-binding antibody, 6F12 (Fig. S4L and S4M). Presumably, viral aggregation in the presence of head-binding antibodies, but not stalk-binding antibodies, is due to antibody epitope access. Head-binding antibodies would be expected to have access to the head domains of HA proteins on nearby viruses, while stalk-binding epitopes are buried near the viral membrane, precluding access. Remarkably, binding of what appear to be individual antibody molecules on viral spikes is observed in tomogram slices (Fig. S4J and S4K, red arrowheads).

### The stalk-binding antibody 6F12 binds conformational epitope and induces rearrangement in HA stalk residues.

**FIG 5:**
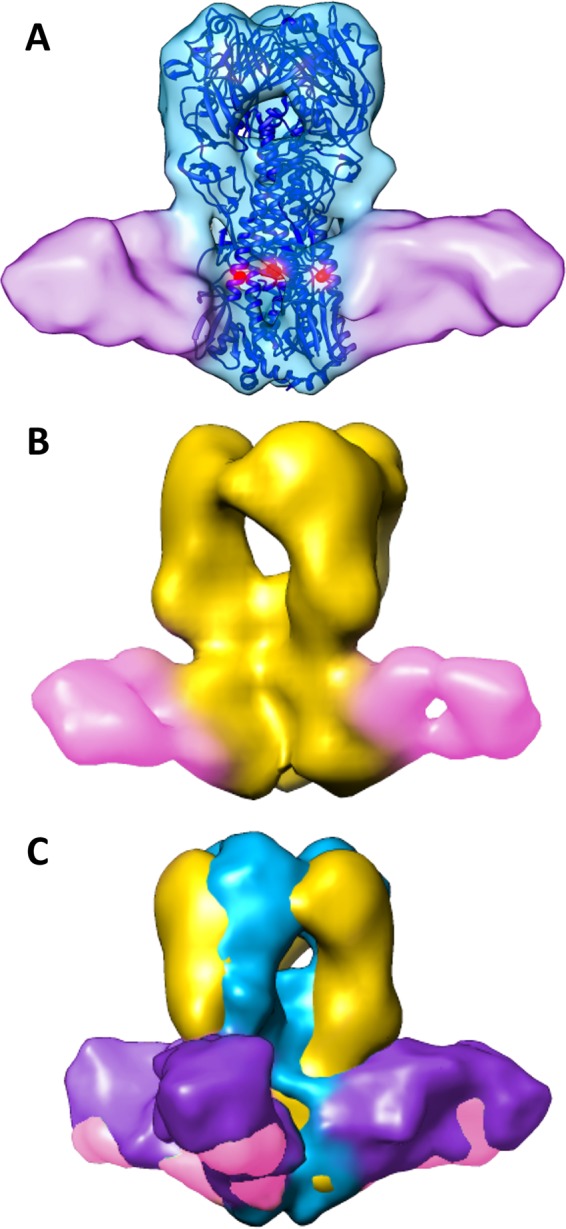
Structures of HA bound to antibody 6F12 suggest that the stalk epitope is retained in chimeric HA. (A) An isosurface representation of H1 HA (cyan) bound to antibody 6F12 (purple) is shown fitted with the H1 HA crystal structure. Residues identified previously as escape mutants (20) are shown as red spheres. (B) The structure of cH5/1 HA (gold) is shown bound to 6F12 (pink). (C) 6F12-bound cH5/1 (gold) and 6F12-bound H1 (cyan) HA structures are aligned at the stalk region. The rotation in the cH5/1 structure is apparent in the offset of the head domains of each HA map.

Antibody 6F12 targets the stalk region of H1 HA (20), and as expected, it bound to both H1 (Fig. 5A) and cH5/1 HA (Fig. 5B). The structure of 6F12-bound cH5/1 HA maintains a 60° rotation between the head and stalk regions (Fig. 5C), consistent with the twist in the unbound chimeric form. The 6F12 epitope is conformational in nature (6) and dependent on the positions of residues in both the HA1 and HA2 portions of the stalk (16–18, 25). The structure of H1 HA in complex with 6F12 supports this claim: extra density apparent in the unbound H1 and H5 HA maps (Fig. 1C, black asterisks) corresponds with the locations of carbohydrate moieties present at the glycosylation sites on the tip of HA1 and are shifted in comparison to their locations in H1 HA (see Fig. S2 in the supplemental material). Rearrangement in the stalk portion of HA upon 6F12 binding has been predicted due to the location of the single escape mutation generated for this antibody, residue 44 of the HA2 subunit (20) (Table S2), which is found in the inner portion of the HA2 alpha helices (highlighted red residues in Fig. 5A).

An important difference between the chimeric virus and the nonchimeric strains is that even at high antibody concentrations, ~40% of the cH5/1 HA proteins resolved in subvolume average classes remained unbound by 6F12 antibody (see Fig. S3 in the supplemental material). In contrast, there was no detectable fraction of unbound HA proteins from pH1N1 viruses under similar conditions of antibody incubation. This disparity occurs despite the fact that both proteins show similar high binding affinities for 6F12 (Table S4). Therefore, incomplete binding of 6F12 to cH5/1 HA may be explained by the close packing of glycoproteins on the surfaces of cH5/1N1 viruses (Fig. 3), where access to the glycoprotein may be more limited than on the surfaces of pH1N1 viruses. A comparison of virus neutralization by 6F12 IgG suggests that pH1N1 viruses are 5-fold more susceptible to *in vitro* neutralization by 6F12 than cH5/1N1 viruses are (Table S3), indicating that the efficacy of viral neutralization by 6F12 may correlate with its accessibility to HA on the viral surface.

Influenza viruses bearing chimeric HA proteins such as cH5/1 and related variants are set to undergo human clinical trials as part of a universal influenza vaccine candidate. Our results show that cH5/1 HA expressed on the viral surface displays a 60° rotation between the stalk and head domains compared to the native H1 or H5 proteins. Despite this remarkable difference compared to wild-type HAs, however, the protein remains functional, supports growth of infectious virus to high titers, and retains the ability to bind neutralizing antibodies that target both the head and stalk. While there is no evidence to suggest that the unique architecture of cH5/1 HA plays a significant role in the induction of broadly neutralizing antibodies, characterization of this vaccine candidate reveals an astonishing degree of structural plasticity of the functional HA molecule. Importantly, our data suggest that the stalk domain retains its conservation of broadly neutralizing stalk epitopes despite radical structural changes to the molecule or antigenic drift of the head domain, highlighting its potential as a long-lasting universal influenza virus vaccine.

## MATERIALS AND METHODS

### Virus purification.

Viruses were grown in 10-day-old specific-pathogen-free embryonated chicken eggs (Charles River) for 48 h at 37°C. Allantoic fluid was harvested and subjected to low-speed centrifugation (relative centrifugal force [RCF] of 3,000 for 30 min at 4°C) to remove cellular debris. Viruses were pelleted through a 30% sucrose cushion (30% sucrose in NTE buffer [100 mM NaCl, 10 mM Tris-HCl, 1 mM EDTA] [pH 7.4]) by ultracentrifugation (25,000 rpm for 2 h at 4°C using a Beckman SW28 rotor). After aspiration of the supernatant, virus pellets were resuspended in phosphate-buffered saline (PBS). When necessary, virus preparations were inactivated with 0.03% formalin for 48 h at 4°C.

### Generation of escape mutations.

Either A/Vietnam/1203/2004 (low path 6:2 reassortant with the polybasic cleavage site removed) or A/Netherlands/602/2009 virus was first diluted to a concentration of 1 × 10^6^ PFU/µl in PBS. One hundred microliters of the virus was mixed with 50 µg of monoclonal antibody (either 3F5 or 7B2, respectively), and after incubation at room temperature (RT) for 1 h, the entire volume was injected into 8-day-old specific-pathogen-free embryonated chicken eggs and allowed to grow for 48 h at 37°C. The presence of virus was confirmed using a hemagglutinin (HA) assay. Allantoic fluid was harvested and subjected to low-speed centrifugation (relative centrifugal force of 3,000 for 30 min at 4°C) to remove cellular debris. Viruses were plaqued on Madin-Darby canine kidney (MDCK) cells, and plaques were picked and grown up again in embryonated eggs. RNA was extracted from the allantoic fluid using TRIzol reagent (Invitrogen). cDNA was generated using Superscript III reverse transcriptase (Invitrogen), and HA segments were subjected to Sanger sequencing. Identified escape mutations were introduced into the respective H1 or H5 expression vectors, and escape was confirmed by showing loss of binding by immunofluorescence microscopy as described previously (26).

### Microneutralization assays.

MDCK cells were seeded onto a 96-well plate at 1.5 × 10^4^ to 1.8 × 10^4^ cells per well and incubated at 37°C overnight. Antibodies were diluted in PBS to a starting concentration of 10 µg/50 µl and then 3-fold diluted on a 96-well plate. Viruses were diluted to a concentration of 100 PFU per 50 µl in infection medium (1× minimal essential medium [MEM] plus tosylsulfonyl phenylalanyl chloromethyl ketone [TPCK]-treated trypsin at 1:1,000). Fifty microliters of diluted virus was incubated with 50 µl of serially diluted antibody for 1 h at room temperature. The cells were washed with PBS, and 100 µl of virus-antibody mixture was transferred onto the cells. The cells were incubated at 37°C for 1 h. The cells were then washed, and 50 µl of serially diluted antibody and 50 µl of infection medium were added to each well. After 24 h of incubation at 37°C, the cells were washed with PBS, fixed with ice-cold 80% acetone, and moved to −20°C for at least 1 h. The plates were washed with 1× PBS plus 0.1% Tween 20 (PBS-T), and 100 µl of 3% hydrogen peroxide was added to each well. After 30 min, the hydrogen peroxide was replaced with 200 µl of PBS-T plus 3% milk (blocking solution). After 30 min, the blocking solution was removed, and 50 µl of a biotinylated mouse monoclonal antinucleoprotein (anti-NP) antibody (MAB8257B; EMD Millipore) diluted 1:2,000 in blocking solution was added to each well. After 1 h, the plates were washed, and 50 µl of horseradish peroxidase (HRP)-conjugated streptavidin (EMD Millipore) diluted 1:5,000 in blocking solution was added to each well. After 1 h, the plates were washed and developed with 100 µl of SigmaFast OPD (*o*-phenylenediamine dihydrochloride) substrate (Sigma-Aldrich) for 30 min and stopped with 50 µl of 3 M hydrochloric acid. The plates were then read at an optical density (OD) of 490. Fifty percent inhibitory concentrations (IC_50_s) were calculated in GraphPad Prism.

### Biolayer interferometry.

Purified baculovirus-expressed hemagglutinin was first diluted to 1.5 mg/ml (0.100 ml) with 1× PBS and biotinylated (EZ-Link NHS-PEG4-biotin [NHS stands for *N*-hydroxysuccinimide ester, and PEG4 stands for a 4-unit polyethylene glycol group]; Thermo Fisher Scientific, Inc.) for 30 min at RT. Nonreacted biotin was removed by buffer exchange using Zeba spin desalting columns (Thermo Fisher Scientific, Inc.) and spun at 1,500 RCF for 1 min. Biolayer interferometry (BLI) was performed using an Octet Red 96 system (ForteBio, Inc.). Assays were performed in solid black 96-well plates using streptavidin biosensors (ForteBio, Inc.). All purified HAs and monoclonal antibodies (MAbs) were diluted in kinetics buffer (1× PBS containing 0.02% Tween 20 and 0.1% bovine serum albumin [BSA]). The biotinylated HAs were initially diluted to 20 µg/ml; the MAbs were diluted to a starting concentration of 100 µg/ml and subsequently serially diluted threefold. The streptavidin sensors were washed for 180 s prior to loading with biotinylated HAs for 300 s. The sensors were washed again for 180 s before association with MAbs for 300 s. The dissociation step was applied for 900 s. Experimental data were fit with the 1:1 binding model, and the data set was analyzed with global fitting using Octet software to calculate the *K_D_* (equilibrium dissociation constant), *k_a_* (association constant), and *k*_dis_ (dissociation constant)*.*

### Quantification of the NA/HA ratio on the viral surface.

One hundred nanograms of purified pH1N1 or purified cH5/1N1 virus was run on a polyacrylamide gel (5 to 20% gradient; Bio-Rad) under nonreducing conditions and stained overnight using Coomassie blue G-250 (SimplyBlue SafeStain; Invitrogen). The gel was washed three times with heated distilled H_2_O (dH_2_O) to remove background staining. Sodium dodecyl sulfate-polyacrylamide gel electrophoresis (SDS-PAGE) band quantification was performed using the densitometry function of the Java-based image processing program, ImageJ. HA and neuraminidase (NA) bands were identified by molecular weight and matched to corresponding densitometry peaks produced by the ImageJ software. The area under the peaks (a quantitative estimate of the band density) corresponding to the HA and NA were approximated by the ImageJ program, and the ratio of NA to HA was calculated using these values.

### Preparation of cryo-EM grids.

Purified influenza virus strains expressing either pH1N1, H5N1, or cH5/1N1 glycoproteins were incubated on ice with or without antibodies for 30 min at an approximate ratio of 3 µg antibody to 1 µg virus. Immediately before grid preparation, 10-nm protein A gold was added to the sample, and the mixture was then pipetted onto plasma-cleaned 200-mesh Quantifoil Multi-A carbon grids (Quantifoil). Using a Leica EM grid plunger (Leica Microsystems), excess buffer was blotted at room temperature and 95% humidity, and the grids were plunge-frozen in liquid ethane maintained at about −180°C. The grids were stored in liquid nitrogen until use.

### Cryo-electron microscopy.

Specimens were imaged in a Titan Krios transmission electron microscope (FEI Company) operated at 300 kV and equipped with a GIF Quantum energy filter (Gatan) at a slit width of 20 eV. Images were recorded on a K2 Summit camera (Gatan) at a pixel size of 2.2 Å. Tilt series were collected at ±60° in 2° increments at a magnification of ×64,000 and a defocus range of 2 to 3 µm. The total dose used was approximately 120 e^−^/Å^2^.

### Tomographic image analysis.

Fiducial-based reconstruction of tomograms using weighted back-projection techniques was performed as previously described (23, 27). Typically, between 9 and 20 tilt series were collected for each influenza virus complex (see Table S5 in the supplemental material). Glycoprotein spikes were picked for subvolume averaging either automatically, as previously described (22, 23, 28) or by manually selecting spike locations (Table S5). Alignment, classification, and 3D averaging of the subvolumes were performed as previously described (22, 29). Briefly, subvolumes were subjected to successive rounds of alignment and classification until particles converged into multiple similar structural classes of approximately 100 particles each. A representative map from each data set was selected for presentation. A class size of 100 subvolumes was chosen in order to distinguish heterogeneity within the data. Noisy or misaligned particles were discarded at each iteration during refinement. Because the initial selection of spikes is done without bias to the effect of the missing wedge and other features that introduce noise in the tomogram, there is a large attrition between initially picked volumes and those that meet the criteria for inclusion in the final map. Because of the small numbers of spike volumes that make up the density maps, Fourier shell correlation (FSC) plots are not reliable ways to measure resolution. However, we estimate the average resolution of all maps presented to be ~25 Å based on comparison with features observed when X-ray structures are filtered to this resolution.

### Viral surface area, glycoprotein density, and interspike distance measurements.

Viral surface area (SA) was calculated from tomographic reconstructions of four individual virions from each viral strain (pH1N1, cH5/1N1, and H5N1) using UCSF Chimera software. Viruses with clearly visible tomographic top views of the trimeric HA protein were selected. To calculate SA, virion regions of interest bounded by a length (*h*) and a width (*l*_arc_) were modeled as cylinders with the virus diameter (*d*_1_). The diameter (*d*_1_) of each virion was measured at a tomogram slice in the center of the representative virion. The diameter (*d*_2_) and length (*h*) of the tomographic slice of the region of interest containing visible HA top views was measured. The following equations were used for SA calculations, where *l*_arc_ is the length of the arc of the specified viral segment: SA = *l*_arc_ × *h*, where *l*_arc_ = θ × *d*_1_ and θ = sin^−1^((*d*_2_)/(*d*_1_)). The number of HA spikes within each region of interest was counted, and spike density (per 100 nm^2^) for each virus strain was determined based on the SA calculation. Distances between nearest neighbor spikes were measured by determining the distance between a point at the center of each spike. All measurements were taken in IMOD and scaled by the camera pixel size (2.2 Å/pixel) and tomogram binning factor of 8. Statistical significance between the mean values was determined by one-way analysis of variance (ANOVA) using GraphPad Prism software.

### Crystal structure fitting.

The Protein Data Bank identifiers (IDs) of the H1 and H5 crystal structures fit into isosurface representations of HA were 3LZG (30) and 2FK0 (31), respectively.

### Model of glycoprotein spike distribution.

Virus diameter was measured at the center of each filamentous virus for all influenza virus strains studied using UCSF Chimera software. A theoretical value for the number of HA spikes per 1 µm virus (theoretically modeled by a cylinder with hemispheres on each end) was calculated based on the average measured virus diameter and glycoprotein density for each strain. A model influenza virus was created for each strain using the segmented viral density from a representative tomogram using Cinema 4D. Isosurface representations of the glycoprotein density maps were distributed on the model virus surface according to the strain-specific calculated densities.

### Accession number.

Cryo-EM density maps for the reported structures have been deposited in EMDataBank (www.emdatabank.org) and given accession numbers EMD-6607 to EMD-6614.

## SUPPLEMENTAL MATERIAL

Figure S1  Chimeric HA structure shows rotation between the stalk and head domains compared to H1 or H5 HA. (A and B) Overlays between H5 HA and either cH5/1 HA (A) or H1 HA (B) are shown. Cross-sectional images taken at the stalk (bottom inset panel) or head (top inset panel) are shown for each overlay. Download Figure S1, PDF file, 0.6 MB

Figure S2  Structure of H1 HA bound to stalk-binding 6F12 antibody suggests that antibody binding induces movement within HA1. An isosurface representation of the H1 HA protein bound to the stalk-binding 6F12 antibody (HA ectodomain shown in cyan, antibody shown in purple) is shown overlaid with the unbound H1 HA structure (gray). Comparison of the two maps reveals movement of the glycosylated tip of HA1 when antibody 6F12 is bound (boxed region). A magnified view of the boxed region is shown in the inset. A red arrow indicates movement in the HA1 tip in the 6F12-bound HA structure (cyan) compared to the unbound H1 HA structure (gray). Download Figure S2, PDF file, 0.1 MB

Figure S3  Chimeric HA shows incomplete binding for stalk-binding antibody. Slices through subtomogram class averages in the direction perpendicular to the spike axis are shown for cH5/1N1 incubated with antibody 6F12. Slices progress through the spike from the stalk (left) to the head (right). An unbound class showing only trimeric HA spike density is shown in the top row, while the bottom row shows an antibody-bound class that displays extra density extending out from the stalk region of the spike. Download Figure S3, PDF file, 1.7 MB

Figure S4  Aggregation phenotype is elicited by head-binding, but not stalk-binding, antibodies. (A to C) Images of pH1N1 (A), cH5/1N1 (B), and H5N1 (C) viruses after incubation with head-binding antibodies show aggregation of the viruses. (D to F) Enlarged images of the regions boxed in red in panels A to C are shown. Gold particles (black dots) are seen localized with viruses. (G to I) Viral aggregation is not observed after incubation with an H1-specific, stalk-binding antibody, 6F12 (G and H) or an H5-specific, stalk-reactive antibody, KB2 (I). (J and K) Tomographic slices through cH5/1N1 virions after incubation with 3F5 (J and K) highlight antibody cross-linking (red box in panel J) and visible binding of individual antibody molecules (red arrowheads in panels J and K). (L and M) Extra density is not seen linking spikes on separate viruses when cH5/1N1 is incubated with the stalk-binding antibody, 6F12. Bars, 500 nm (A to C and G to I), 250 nm (D to F), and 50 nm (J to M). Download Figure S4, PDF file, 2.2 MB

Table S1  Virion morphology and glycoprotein density. The average virion diameter (in nanometers), filamentous virion length (in nanometers), interspike distance (in nanometers), and spike density (glycoprotein spikes/100 nm^2^ of viral surface) are given with the respective standard deviations. The statistical significance of measured values was determined by one-way ANOVA (GraphPad Prism). For each row, values that are significantly different from one another are indicated with different letters. Average viral diameter and glycoprotein density values were used to calculate the approximate number of glycoprotein spikes on theoretical filamentous or spherical virions. Table S1, PDF file, 0.1 MB

Table S2  Antibody escape mutants. Locations of residues whose mutation resulted in a loss of antibody binding are listed for 7B2 (anti-H1 HA head), 3F5 (anti-H5 head), or 6F12 (anti-H1 HA stalk) (20). Table S2, PDF file, 0.1 MB

Table S3  Antibody neutralization. Fifty percent inhibitory concentration (IC_50_) values (expressed in micrograms per milliliter) were determined via microneutralization assays for each antibody-HA complex in this study. Table S3, PDF file, 0.1 MB

Table S4  Antibody binding affinity. The equilibrium dissociation constant (*K_D_*) (in molar) of each antibody-HA complex in our study was determined via biolayer interferometry. Table S4, PDF file, 0.04 MB

Table S5  Tomographic image analysis. For each data set, the number of tilt series, the total number of particles picked (manually or automatically), and the number of particles that contributed to the density maps presented here are shown. Table S5, PDF file, 0.1 MB
