# Supplementary material for: Cryo-electron Microscopy Structures of Chimeric Hemagglutinin Displayed on a Universal Influenza Vaccine Candidate
Source: mBio. 2016 Mar 22;7(2):e00257-16. doi: 10.1128/mBio.00257-16 (PMC4807363; doi:10.1128/mBio.00257-16)
Supplement: Figure S1 — Chimeric HA structure shows rotation between the stalk and head domains compared to H1 or H5 HA. (A and B) Overlays between H5 HA and either cH5/1 HA (A) or H1 HA (B) are shown. Cross-sectional images taken at the stalk (bottom inset panel) or head (top inset panel) are shown for each overlay. Download [file mbo002162733sf1.pdf]

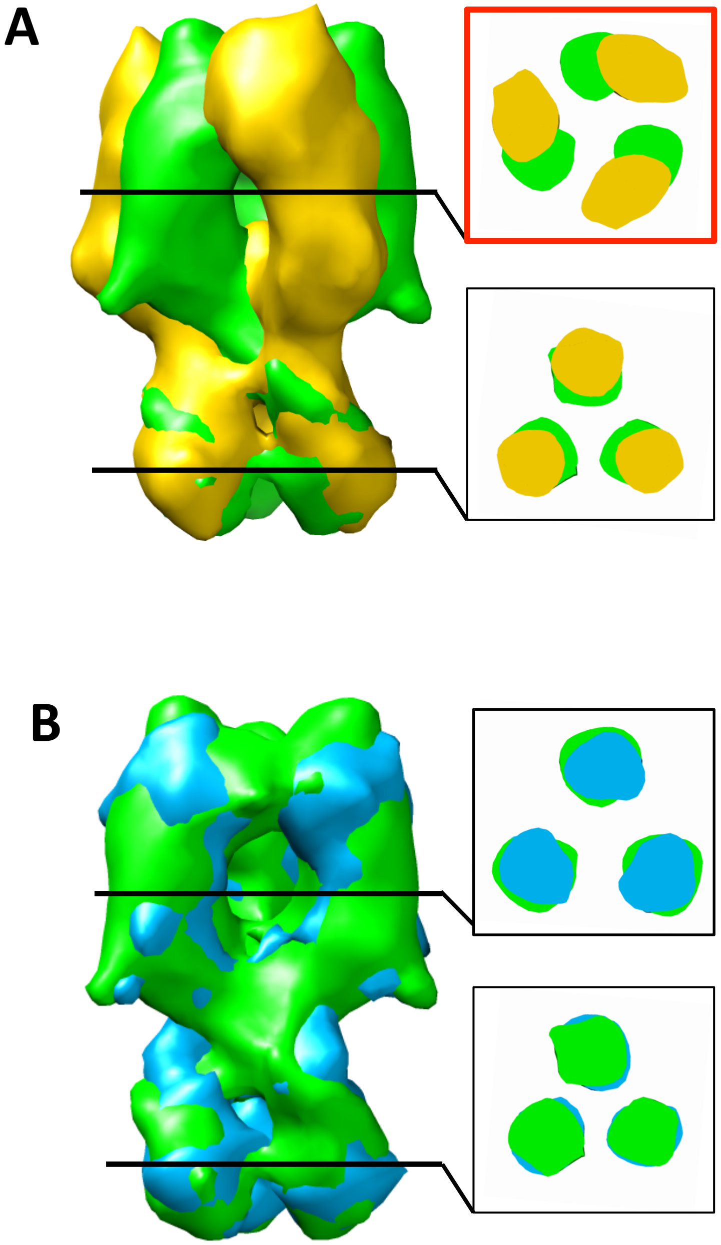

Figure S1. Chimeric HA structure shows rotation between the stalk and head domains compared to H1 or H5 HA. (A, B) Overlays between H5 HA and either cH5/1 HA (A) or H1 HA (B) are shown. Cross-sectional images taken at the stalk (bottom inset panel) or head (top inset panel) are shown for each overlay.
