# Supplementary material for: Cryo-electron Microscopy Structures of Chimeric Hemagglutinin Displayed on a Universal Influenza Vaccine Candidate
Source: mBio. 2016 Mar 22;7(2):e00257-16. doi: 10.1128/mBio.00257-16 (PMC4807363; doi:10.1128/mBio.00257-16)
Supplement: Figure S2 — Structure of H1 HA bound to stalk-binding 6F12 antibody suggests that antibody binding induces movement within HA1. An isosurface representation of the H1 HA protein bound to the stalk-binding 6F12 antibody (HA ectodomain shown in cyan, antibody shown in purple) is shown overlaid with the unbound H1 HA structure (gray). Comparison of the two maps reveals movement of the glycosylated tip of HA1 when antibody 6F12 is bound (boxed region). A magnified view of the boxed region is shown in the inset. A red arrow indicates movement in the HA1 tip in the 6F12-bound HA structure (cyan) compared to the unbound H1 HA structure (gray). Download [file mbo002162733sf2.pdf]

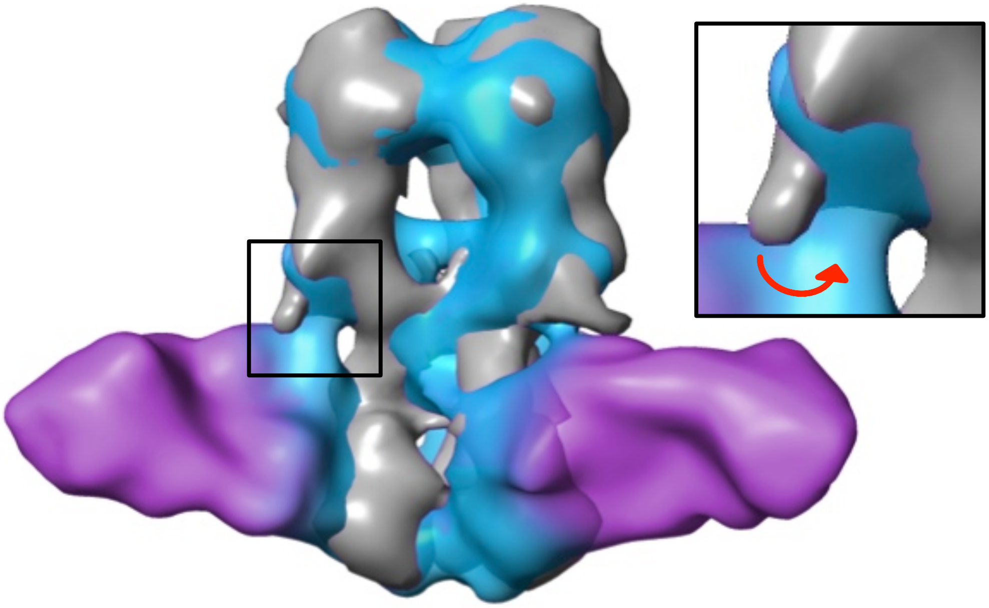

Figure S2. Structure of H1 HA bound to stalk-binding 6F12 antibody suggests that antibody binding induces movement within HA1. An isosurface representation of the H1 HA protein bound to the stalk-binding 6F12 antibody (HA ectodomain in cyan, antibody in purple) is shown overlaid with the unbound H1 HA structure (grey). Comparison of the two maps reveals movement of the glycosylated tip of HA1 when 6F12 is bound (boxed region). A magnified view of the boxed region is shown in the inset. A red arrow indicates movement in the HA1 tip in the 6F12-bound HA structure (cyan) compared to the unbound H1 HA structure (grey).
