# Supplementary material for: Cryo-electron Microscopy Structures of Chimeric Hemagglutinin Displayed on a Universal Influenza Vaccine Candidate
Source: mBio. 2016 Mar 22;7(2):e00257-16. doi: 10.1128/mBio.00257-16 (PMC4807363; doi:10.1128/mBio.00257-16)
Supplement: Figure S3 — Chimeric HA shows incomplete binding for stalk-binding antibody. Slices through subtomogram class averages in the direction perpendicular to the spike axis are shown for cH5/1N1 incubated with antibody 6F12. Slices progress through the spike from the stalk (left) to the head (right). An unbound class showing only trimeric HA spike density is shown in the top row, while the bottom row shows an antibody-bound class that displays extra density extending out from the stalk region of the spike. Download [file mbo002162733sf3.pdf]

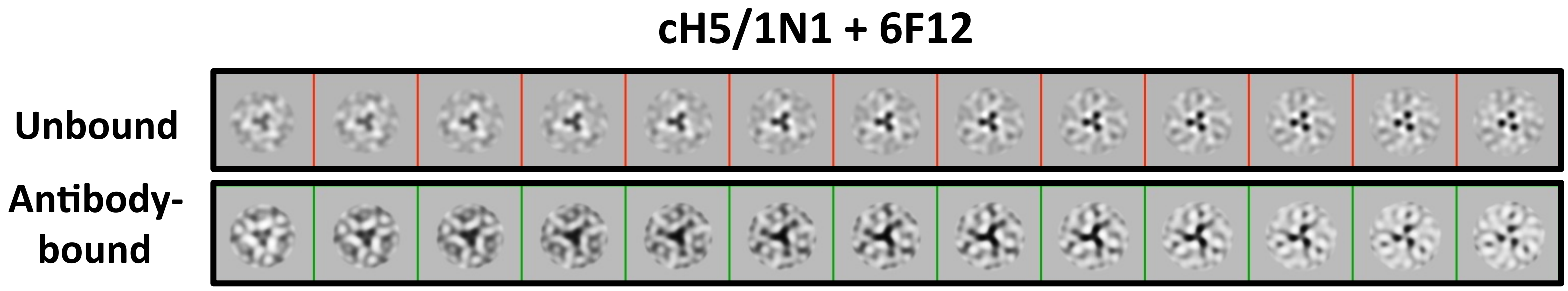

Figure S3. Chimeric HA shows incomplete binding for stalk-binding antibody. Slices through subtomogram class averages in the direction perpendicular to the spike axis are shown for cH5/1N1 incubated with 6F12. Slices progress through the spike from the stalk (left) to the head (right). An unbound class showing only trimeric HA spike density is shown in the top row, while the bottom row shows an antibody-bound class that displays extra density extending out from the stalk region of the spike.
