# Supplementary material for: Cryo-electron Microscopy Structures of Chimeric Hemagglutinin Displayed on a Universal Influenza Vaccine Candidate
Source: mBio. 2016 Mar 22;7(2):e00257-16. doi: 10.1128/mBio.00257-16 (PMC4807363; doi:10.1128/mBio.00257-16)
Supplement: Figure S4 — Aggregation phenotype is elicited by head-binding, but not stalk-binding, antibodies. (A to C) Images of pH1N1 (A), cH5/1N1 (B), and H5N1 (C) viruses after incubation with head-binding antibodies show aggregation of the viruses. (D to F) Enlarged images of the regions boxed in red in panels A to C are shown. Gold particles (black dots) are seen localized with viruses. (G to I) Viral aggregation is not observed after incubation with an H1-specific, stalk-binding antibody, 6F12 (G and H) or an H5-specific, stalk-reactive antibody, KB2 (I). (J and K) Tomographic slices through cH5/1N1 virions after incubation with 3F5 (J and K) highlight antibody cross-linking (red box in panel J) and visible binding of individual antibody molecules (red arrowheads in panels J and K). (L and M) Extra density is not seen linking spikes on separate viruses when cH5/1N1 is incubated with the stalk-binding antibody, 6F12. Bars, 500 nm (A to C and G to I), 250 nm (D to F), and 50 nm (J to M). Download [file mbo002162733sf4.pdf]

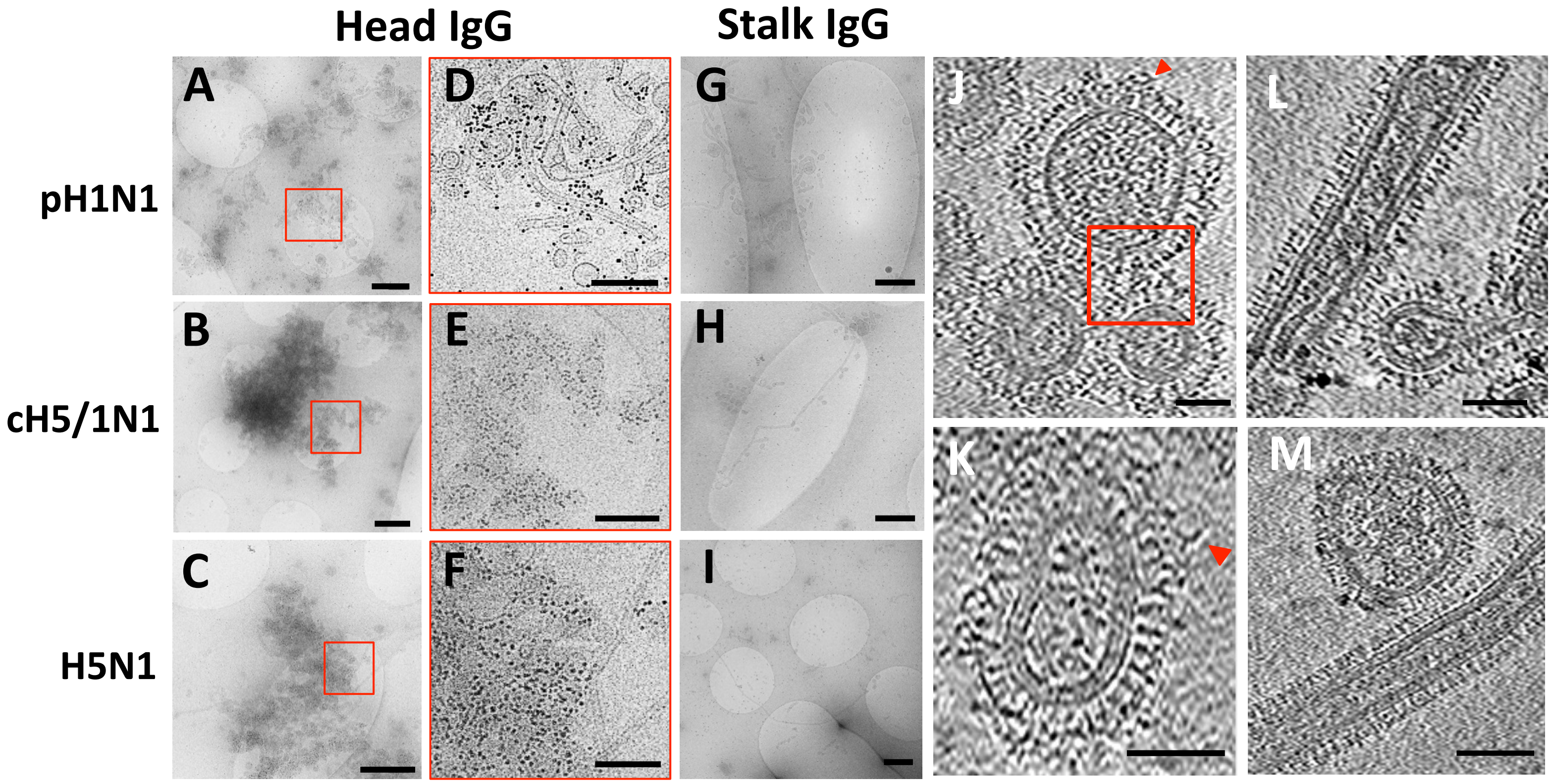

Figure S4. Aggregation phenotype is elicited by head-binding, but not stalk-binding, antibodies. Images of pH1N1, cH5/1N1 and H5N1 viruses after incubation with head-binding antibodies (A-C, respectively) show aggregation of the viruses. Enlarged images of the regions boxed in red are shown in (D-F). Gold particles (black dots) are seen localized with viruses. Viral aggregation is not observed after incubation with an H1-specific, stalk-binding antibody, 6F12 (G, H) or an H5-specific, stalk-reactive antibody, KB2 (I). Tomographic slices through cH5/1N1 virions after incubation with 3F5 (J, K) highlight antibody cross-linking (J, red box) and visible binding of individual antibody molecules (J & K, red arrowheads). Extra density is not seen linking spikes on separate viruses when cH5/1N1 is incubated with the stalk-binding antibody, 6F12 (L, M). Scale bars indicate 500 nm in panels (A-C) and (G-I), 250 nm in panels (D-F) and 50 nm in panels (J-M).
