# Supplementary material for: Cryo-electron Microscopy Structures of Chimeric Hemagglutinin Displayed on a Universal Influenza Vaccine Candidate
Source: mBio. 2016 Mar 22;7(2):e00257-16. doi: 10.1128/mBio.00257-16 (PMC4807363; doi:10.1128/mBio.00257-16)
Supplement: Table S1 — Virion morphology and glycoprotein density. The average virion diameter (in nanometers), filamentous virion length (in nanometers), interspike distance (in nanometers), and spike density (glycoprotein spikes/100 nm2 of viral surface) are given with the respective standard deviations. The statistical significance of measured values was determined by one-way ANOVA (GraphPad Prism). For each row, values that are significantly different from one another are indicated with different letters. Average viral diameter and glycoprotein density values were used to calculate the approximate number of glycoprotein spikes on theoretical filamentous or spherical virions. [file mbo002162733st1.pdf]

	pH1N1	H5N1	cH5/1N1
Average virion diameter (nm)	65.5 ± 15.4[a]	58.5 ± 11.2[a]	65.2 ± 17.5[a]
Average filamentous virion length (nm)	249.3 ± 150.5[a]	253.5 ± 149.3[a]	190.5 ± 100.9[a]
Average interspike distance (nm)	9.7 ± 0.3[a]	10.1 ± 0.5[a]	8.7 ± .03[b]
Average spike density (spikes/100 nm^2)	1.0 ± 0.2[a]	0.9 ± 0.1[a]	1.6 ± 0.2[b]
Average number of trimeric spikes per filamentous virion*	1975	1691	3213
Average number of trimeric spikes per spherical virion**	302	289	493

[a,b] Indicate values that are significantly different
*1 μm in length
** Radius of 50 nm

Table S1. Virion morphology and glycoprotein density. Average virion diameter (nm), filamentous virion length (nm), interspike distance (nm), and spike density (glycoprotein spikes/100 nm^2 of viral surface) are given with respective standard deviations. Statistical significance of measured values was determined by one-way ANOVA (GraphPad Prism). For each row, values that are significantly different from one another are indicated with different letters. Average viral diameter and glycoprotein density values were used to calculate the approximate number of glycoprotein spikes on theoretical filamentous or spherical virions.
