# Supplementary material for: Cryo-electron Microscopy Structures of Chimeric Hemagglutinin Displayed on a Universal Influenza Vaccine Candidate
Source: mBio. 2016 Mar 22;7(2):e00257-16. doi: 10.1128/mBio.00257-16 (PMC4807363; doi:10.1128/mBio.00257-16)
Supplement: Table S2 — Antibody escape mutants. Locations of residues whose mutation resulted in a loss of antibody binding are listed for 7B2 (anti-H1 HA head), 3F5 (anti-H5 head), or 6F12 (anti-H1 HA stalk) (20). [file mbo002162733st2.pdf]

**Antibody (Viral Strain)**	**Escape Mutant**	**Reference**
7B2 (NL09)	N142T	This study
3F5 (VN04)	K205N	This study
6F12 (NL09)	A44V	(Tan, *et al*. 2012. *J. Virol*.)

Table S2. Antibody Escape Mutants. Locations of residues whose mutation resulted in a loss of antibody binding are listed for 7B2 (anti-H1 HA head), 3F5 (anti-H5 head) or 6F12 (anti-H1 HA stalk) (Tan. 2012. J Virol).
