# Supplementary material for: Cryo-electron Microscopy Structures of Chimeric Hemagglutinin Displayed on a Universal Influenza Vaccine Candidate
Source: mBio. 2016 Mar 22;7(2):e00257-16. doi: 10.1128/mBio.00257-16 (PMC4807363; doi:10.1128/mBio.00257-16)
Supplement: Table S3 — Antibody neutralization. Fifty percent inhibitory concentration (IC50) values (expressed in micrograms per milliliter) were determined via microneutralization assays for each antibody-HA complex in this study. [file mbo002162733st3.pdf]

	pH1N1	cH5/1N1	H5N1
**7B2**	0.00779	>100	>100
**6F12**	4.889	26.24	>100
**3F5**	>100	0.7228	72.84

Table S3. Antibody neutralization. IC_{50} (50% inhibitory concentration; expressed as μg/ml) values were determined via microneutralization assays for each antibody-HA complex in this study.
