# Supplementary material for: Cryo-electron Microscopy Structures of Chimeric Hemagglutinin Displayed on a Universal Influenza Vaccine Candidate
Source: mBio. 2016 Mar 22;7(2):e00257-16. doi: 10.1128/mBio.00257-16 (PMC4807363; doi:10.1128/mBio.00257-16)
Supplement: Table S4 — Antibody binding affinity. The equilibrium dissociation constant (KD) (in molar) of each antibody-HA complex in our study was determined via biolayer interferometry. [file mbo002162733st4.pdf]

	**pH1N1**	**cH5/1N1**	**H5N1**
**6F12**	1.36E-09	1.25E-09	--
**3F5**	--	1.90E-09	1.44E-09
**7B2**	1.56E-10	--	--

Table S4. Antibody binding affinity. The K_D (M) of each antibody-HA complex in our study was determined via BioLayer Interferometry.
