# Supplementary material for: Cryo-electron Microscopy Structures of Chimeric Hemagglutinin Displayed on a Universal Influenza Vaccine Candidate
Source: mBio. 2016 Mar 22;7(2):e00257-16. doi: 10.1128/mBio.00257-16 (PMC4807363; doi:10.1128/mBio.00257-16)
Supplement: Table S5 — Tomographic image analysis. For each data set, the number of tilt series, the total number of particles picked (manually or automatically), and the number of particles that contributed to the density maps presented here are shown. [file mbo002162733st5.pdf]

Dataset	# Tilt Series	# Picked Particles	# Particles in Map Shown	Manual or Automated Particle Picking
**pH1N1**	9	1832	55	Manual
**cH5/1N1**	27	1927	166	Manual & Automated
**H5N1**	10	1049	129	Manual
**pH1N1 + 7B2 IgG**	15	1446	297	Automated
**cH5/1N1 + 3F5 IgG**	16	662	99	Manual
**H5N1 + 3F5 IgG**	16	1104	100	Manual
**pH1N1 + 6F12 IgG**	10	1137	85	Automated
**cH5/1N1 + 6F12 IgG**	10	681	134	Automated

Table S5. Tomographic image analysis. For each dataset, the number of tilt series, the total number of particles picked (manually or automatically), and the number of particles that contributed to the density maps presented here are shown.
